# Cryo-EM of NHEJ supercomplexes provides insights into DNA repair

**DOI:** 10.1016/j.molcel.2021.07.005

**Published:** 2021-08-04

**Authors:** Amanda K. Chaplin, Steven W. Hardwick, Antonia Kefala Stavridi, Christopher J. Buehl, Noah J. Goff, Virginie Ropars, Shikang Liang, Taiana Maia De Oliveira, Dimitri Y. Chirgadze, Katheryn Meek, Jean-Baptiste Charbonnier, Tom L. Blundell

**Affiliations:** 1 Department of Biochemistry, University of Cambridge, Sanger Building, Tennis Court Road, Cambridge CB2 1GA, UK; 2 CryoEM Facility, Department of Biochemistry, University of Cambridge, Sanger Building, Tennis Court Road, Cambridge CB2 1GA, UK; 3 College of Veterinary Medicine, Department of Microbiology & Molecular Genetics, Department of Pathobiology & Diagnostic Investigation, Michigan State University, East Lansing, MI 48824, USA; 4 Institute for Integrative Biology of the Cell (I2BC), Institute Joliot, CEA, CNRS, Université Paris-Saclay, 91198, Gif-sur-Yvette Cedex, France; 5 AstraZeneca R&D, Discovery Sciences, Mechanistic and Structural Biology, Cambridge, UK; 6 These authors contributed equally; 7 Lead contact

## Abstract

Non-homologous end joining (NHEJ) is one of two critical mechanisms utilized in humans to repair DNA double-strand breaks (DSBs). Unrepaired or incorrect repair of DSBs can lead to apoptosis or cancer. NHEJ involves several proteins, including the Ku70/80 heterodimer, DNA-dependent protein kinase catalytic subunit (DNA-PKcs), X-ray cross-complementing protein 4 (XRCC4), XRCC4-like factor (XLF), and ligase IV. These core proteins bind DSBs and ligate the damaged DNA ends. However, details of the structural assembly of these proteins remain unclear. Here, we present cryo-EM structures of NHEJ supercomplexes that are composed of these core proteins and DNA, revealing the detailed structural architecture of this assembly. We describe monomeric and dimeric forms of this supercomplex and also propose the existence of alternate dimeric forms of long-range synaptic complexes. Finally, we show that mutational disruption of several structural features within these NHEJ complexes negatively affects DNA repair.

## In brief

Non-homologous end joining (NHEJ) is a key mechanism that repairs DNA-double strand breaks. Using cryo-EM, Chaplin et al. present monomeric and dimeric structures of the core NHEJ proteins and reveal their structural architecture. Two long-range synaptic dimers are presented, and mutations of the dimer interfaces negatively affect DNA repair.

## Graphical Abstract

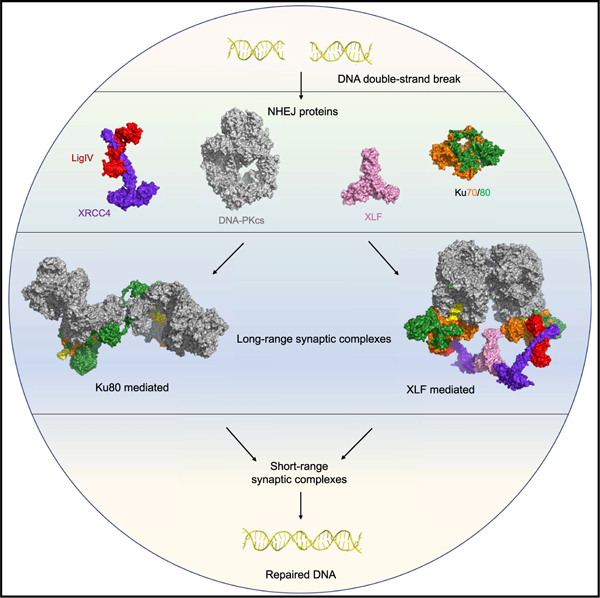

## INTRODUCTION

In all kingdoms of life, the ability to repair DNA damage is essential. Non-homologous end joining (NHEJ) is nearly ubiquitous as a DNA repair mechanism and is one of the two main mechanisms utilized to repair DNA double-strand breaks (DSBs) in humans (Harper and Elledge, 2007). DSBs are one of the most dangerous type of DNA damage and, when left unrepaired, have the potential to lead to apoptosis, tumorigenesis, and cancer (van Gent et al., 2001). Fully understanding the spatial and temporal orchestration of the complex multicomponent process of NHEJ has long been a challenge; however, recent advances in cryoelectron microscopy (cryo-EM) have accelerated the structural understanding of some of the proteins and complexes involved. In NHEJ, DSBs are first recognized by the Ku70/80 heterodimer, which subsequently recruits the DNA-dependent protein kinase catalytic subunit (DNA-PKcs), a large protein kinase belonging to the phosphoinositide-3-kinase-related kinase (PIKK) family (Hartley et al., 1995). Ku70/80, DNA, and DNA-PKcs together form the DNA-PK complex or holoenzyme (Gottlieb and Jackson, 1993; Hartley et al., 1995; Lees-Miller et al., 1990; Suwa et al., 1994; Walker et al., 1985). The cryo-EM structure of the DNA-PK holoenzyme has been solved to 6.6-Å resolution (Sharif et al., 2017; Yin et al., 2017). We recently improved the resolution of the DNA-PK holoenzyme to 3.5-Å resolution following density modification and revealed a dimer of DNA-PK mediated via the C terminus of Ku80, which had not been reported previously (Chaplin et al., 2021). This dimer provided new structural insights into the NHEJ mechanism, and we hypothesized its ability to act as a central stage for further recruitment and regulation of downstream NHEJ proteins.

In addition to the DNA-PK holoenzyme, canonical proteins involved in NHEJ include X-ray cross-complementing protein 4 (XRCC4) and the XRCC4-like factor (XLF), which have been shown to alternate in long helical filaments that are thought to aid in bringing the broken DNA ends together (Brouwer et al., 2016; Hammel et al., 2011; Mahaney et al., 2013; Wu et al., 2011). The final step in NHEJ involves DNA ligase IV (LigIV), which acts to ligate the two broken ends and forms a constitutive complex with XRCC4 known as LX4 (Bryans et al., 1999). It has been proposed that formation of large assemblies is possible in a two-step mechanism, with an initial long-range synaptic complex formed prior to recruitment of LX4 and XLF in a short-range complex (Graham et al., 2016; Wang et al., 2018). To address whether DNA-PK is able to form higher-order multicomponent assemblies, we collected cryo-EM data for a complex of DNA-PK, LX4, and XLF, revealing the presence of NHEJ supercomplexes formed of all six proteins and DNA in monomeric and dimeric states. Here we discuss the molecular interactions within these complexes and the potential roles of alternate long-range synaptic assemblies in NHEJ pathway progression.

## RESULTS

### Cryo-EM structure of DNA-PK bound to LX4 and XLF

**Figure 1.**
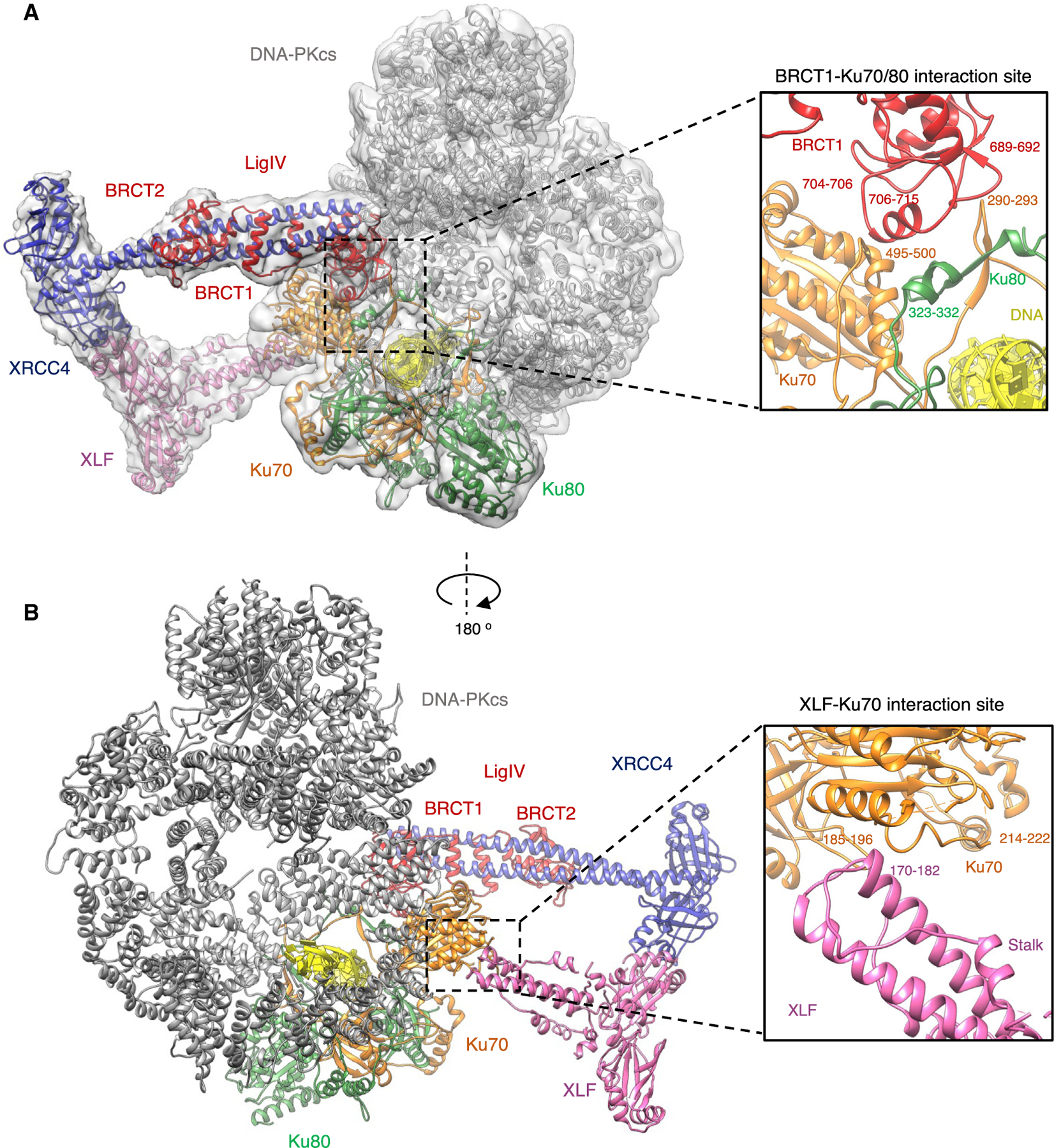
The structure of the assembly of DNA-PK, XRCC4, XLF, and the BRCT tandem repeats of LigIV (A) The overall structure of DNA-PK, XRCC4, XLF, and LigIV BRCT tandem repeats in a monomeric form. DNA-PKcs is shown in gray, Ku70 in orange, Ku80 in green, XLF in pink, XRCC4 in blue, DNA in yellow, and LigIV BRCT repeats in red. The cryo-EM map to 4.3-Å resolution is shown as a gray transparent surface. The inset shows an enlarged view of the interaction between the BRCT1 domain of LigIV (red) and Ku70/80 (orange and green, respectively). (B) The overall structure of DNA-PK, XRCC4, XLF, and LigIV BRCT tandem repeat monomer rotated by 180° and proteins colored according to (A). The inset shows an enlarged view of the interaction between the stalk of XLF (pink) with Ku70 (orange).

To attempt to visualize the higher-order multicomponent assembly, we collected cryo-EM data for a complex of DNA-PK, LX4 (full-length ligase and XRCC4), and full-length XLF using our previously optimized preparation of DNA-PK cryo-EM grids (Chaplin et al., 2021). Following extensive particle classification, we obtained a map of a supercomplex of the core NHEJ proteins and DNA to 4.3 Å resolution (Figure S1). In this map, there is density clearly visible, in which we were able to dock the X-ray crystal structure of XRCC4 with the BRCT (BRCA1 C-terminal) tandem repeats of LigIV(PDB: 3II6) and XLF (PDB: 2R9A) in addition to DNA-PK (PDB: 6ZHA) (Figure S2; Figure 1). Although the construct for LX4 contains full-length LigIV, we do not observe density for the catalytic domain.

### BRCT1 of LigIV interacts with Ku70/80

The major interaction site between DNA-PK and LX4 involves the BRCT1 domain of LigIV and Ku70/80. The interaction of the BRCT1 domain and the Ku70/80 heterodimer has been predicted previously; however, specific sites were not identified (Costantini et al., 2007). The crystal structure of XRCC4 with the BRCT tandem repeats of LigIV can be docked into this additional density of our cryo-EM map with a minor rotation of the BRCT1 domain relative to XRCC4 compared with the X-ray crystal structure (Figure S2F). Even though the cryo-EM map is at moderate resolution in this region, we can define an interaction interface between sections of the BRCT1 domain and the Ku70/80 heterodimer. Specifically, the BRCT1 domain sits in a groove created by the dimer interface between Ku70 and Ku80. The specific interaction sites are between loops of BRCT1 (residues 689–692 and 704–706) with Ku70 (residues 290–293 and 495–500). Sandwiched between these is an interaction between Ku80 residues 323–332 with a helix of the BRCT1 domain of LigIV (residues 706–715) (Figure 1A).

### XLF interactions with XRCC4 and Ku70

The docking of XLF into the cryo-EM map shows a head-to-head interaction with XRCC4 that has been described previously (Hammel et al., 2011; Malivert et al., 2010; Ropars et al., 2011). Additionally, a clear interaction between the stalk of XLF (residues 170–182) and the von Willebrand A (vWA) domain of Ku70 (residues 214–222 and 185–196) is also observed (Figure 1B). We could not, however, observe a direct interaction between XLF and a Ku70 homodimer *in vitro* using isothermal titration calorimetry (data not shown), and we therefore conclude that this is a weak or transient interaction that only occurs when the other core NHEJ proteins are bound. The binding of XLF to Ku70/80 has been the subject of many studies; however, this interaction was thought to be mediated solely by the Ku binding motif (KBM) at the C terminus of XLF and Ku80, with no proven interaction with Ku70 (Grundy et al., 2016; Li et al., 2008; Nemoz et al., 2018; Yano et al., 2008). We did not, however, observe any density corresponding to the flexible C-terminal tail of XLF containing the KBM. Nevertheless, compared with the DNA-PK structure alone (PDB: 6ZHA), the Ku80 vWA domain appears to be in a slightly more open conformation (Figure S3). This may indicate that binding of LX4 and XLF can mediate a movement in this domain in a manner similar to binding of the KBM of XLF to Ku80 (Nemoz et al., 2018). The Ku70 domain remains in the same rotated orientation compared with apo-Ku70/80, as seen previously in the DNA-PK structure (Figure S3; Chaplin et al., 2021).

### XLF mediates formation of an alternative DNA-PK dimer

**Figure 2.**
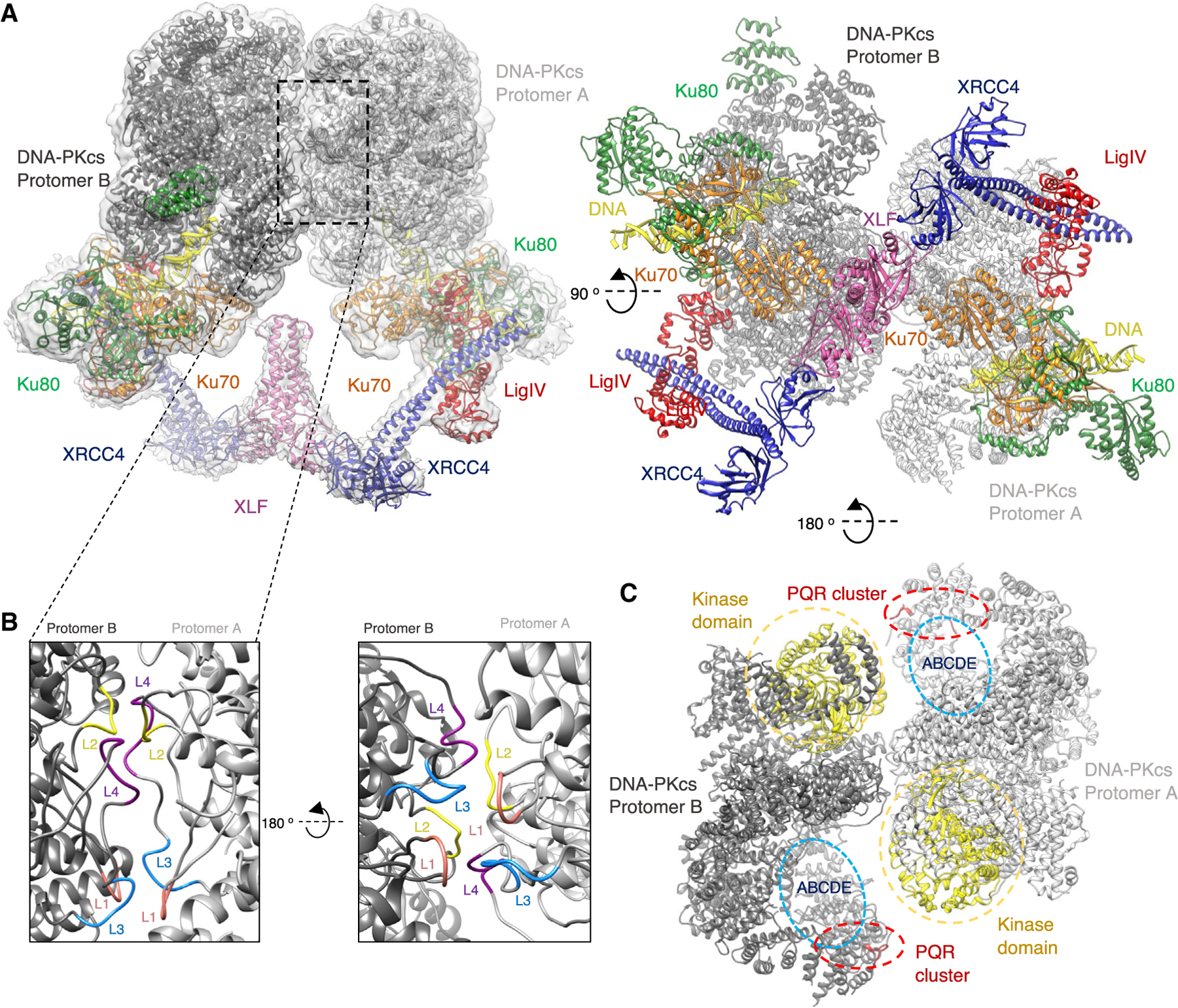
The structure of the dimeric NHEJ supercomplex containing DNA-PK, XRCC4, XLF, and the BRCT tandem repeats of LigIV (A) The overall assembly of the supercomplex XLF-mediated DNA-PK dimer in two orthogonal orientations. DNA-PKcs is shown in gray, Ku70 in orange, Ku80 in green, XLF in pink, XRCC4 in blue, DNA in yellow, and LigIV BRCT repeats in red. The cryo-EM map to 4.1-Å resolution is shown as a gray transparent surface. (B) Two orientations of the dimer interface with loop 1 (L1; residues 898–901) shown in pink, loop 2 (L2; residues 946–950) in yellow, loop 3 (L3; residues 2567–2572) in blue, and loop 4 (L4; residues 2578–2583) in purple, with protomer A in light gray and protomer B in darker gray. (C) A top view of the DNA-PKcs components of the dimer in two shades of gray. The kinase domains (yellow) are shown in close proximity to the PQR (red) and ABCDE (blue) phosphorylation clusters on the opposite protomer (red).

While classifying particles from the DNA-PK-LX4-XLF dataset, we identified a dimeric form of the assembly that differs from the Ku80-mediated DNA-PK dimeric arrangement seen previously (Chaplin et al., 2021). The structure of this new dimer of DNA-PK at 4.1-Å resolution is mediated predominantly via interactions within the circular cradle (Figure 2A). Specifically, the loop of residues 898–901 (loop 1) interacts with the loop of residues 2567–2572 (loop 3) on the opposite protomer. There is a further interaction between residues 946–950 (loop 2) and 2578–2583 (loop 4) (Figure 2B). The arrangement of this new dimer would allow the PQR and possibly the ABCDE phosphorylation clusters on one protomer to be in close proximity to the kinase domain on the opposite protomer. Although the distance between the PQR phosphorylation cluster and the kinase domain cannot be measured confidently, an approximate decrease of ~20 Å is estimated from the monomeric to the dimeric supercomplex structure. The distance of the kinase domain to the ABCDE cluster cannot be measured precisely because the ABCDE loop is disordered. The juxtaposition of phosphorylation and kinase sites within the dimer may explain how these residues are phosphorylated when they are distant from the kinase active site within the DNA-PKcs monomer (Figure 2C; Figure S4). It is also apparent that the LX4 and XLF interactions with the DNA-PK monomer are largely maintained in this dimeric form, with a single XLF homodimer positioned along the dimer axis (Figure S4; Figure 2a). This central positioning of XLF allows it to interact with the vWA domains of Ku70 in both protomers. Remarkably, the arrangement of XRCC4-XLF-XRCC4 within this structure closely resembles the filament arrangement of these proteins as observed from X-ray crystal structures (Hammel et al., 2011; Mahaney et al., 2013; Wu et al., 2011). Compared with the monomeric supercomplex structure, XLF appears to have rotated to enable head-to-head interactions with both XRCC4 homodimers (Figure S5). We see this dimeric supercomplex form only in samples containing LX4 and XLF. A further dataset with XLF omitted showed density for LX4 bound to DNA-PK, but the new DNA-PK dimer form was not observed, indicating that the presence of XLF is crucial for formation of this alternative XLF-mediated DNA-PK dimer (Figure S6).

### Two long-range synaptic DNA-PK dimer complexes

**Figure 3.**
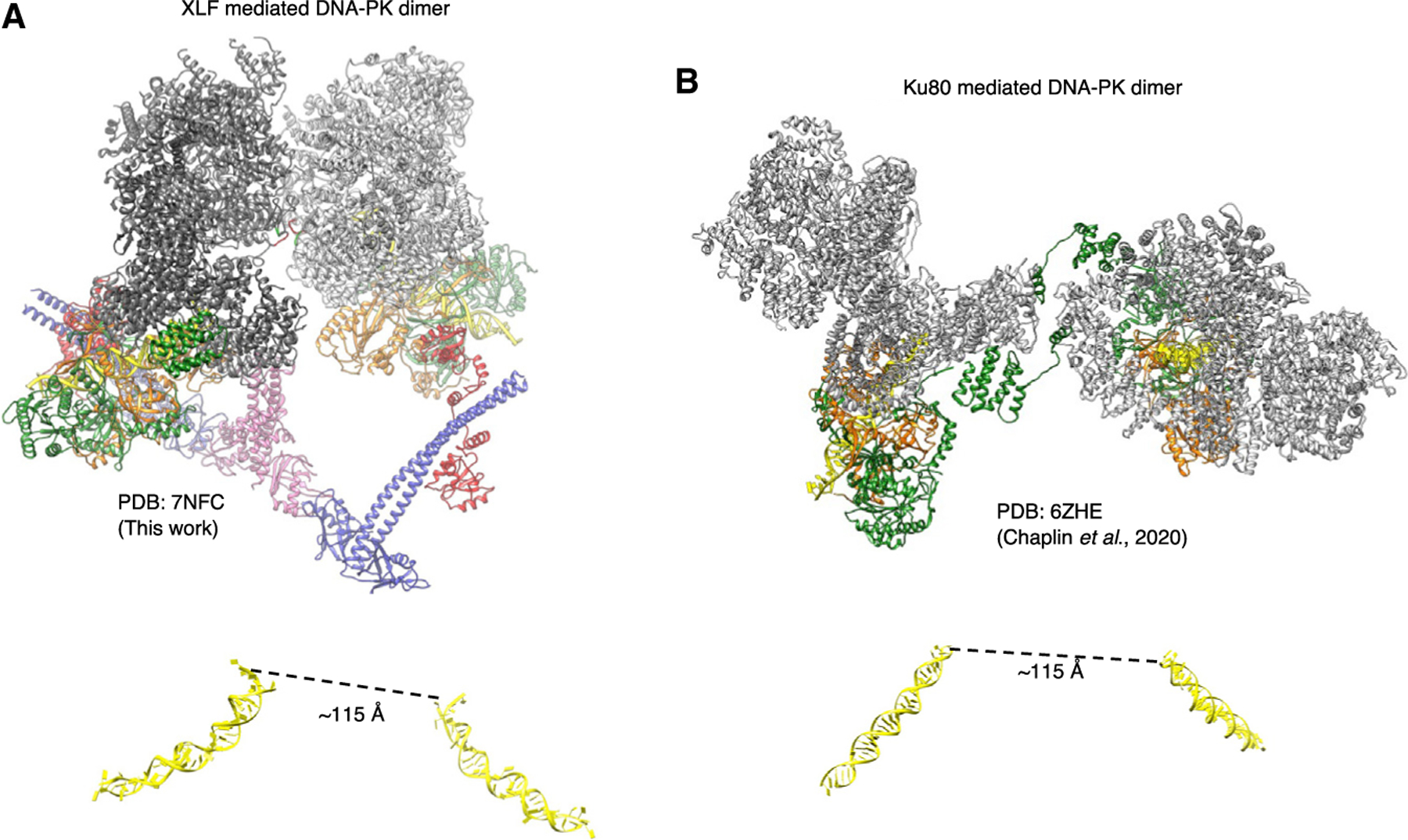
Long-range synaptic complexes (A) NHEJ supercomplex dimer (XLF-mediated DNA-PK dimer [PDB: 7NFC], this work). DNA-PKcs is shown in gray, Ku70 in orange, Ku80 in green, XLF in pink, XRCC4 in blue, DNA in yellow, and LigIV BRCT repeats in red. The DNA is shown below the structure in yellow, with the distance between the DNA ends indicated. (B) The Ku80-mediated DNA-PK dimer (Chaplin et al., 2021) with DNA-PKcs is shown in gray, Ku70 in orange, Ku80 in green, and the DNA in yellow. The DNA is shown in yellow below the structure, with the distance between the DNA ends indicated.

The distance between the two DNA double-strand ends in the XLF-mediated dimeric supercomplex is ~115 Å (Figure 3A); this compares exceptionally well with the previously measured distance between the two DNA ends in the proposed long-range synaptic dimer complex (Graham et al., 2016) as measured by single-molecule fluorescence resonance energy transfer (FRET). Strikingly, the distance between the two DNA ends in the previously published Ku80-mediated dimer (Chaplin et al., 2021) is also exactly ~115 Å (Figure 3B). Because this distance has been proposed previously to represent the arrangement of DNA within long-range synaptic complexes, we propose that the two distinct DNA-PK dimers represent alternate forms of the long-range synaptic complexes.

### Disruption of both long-range DNA-PK dimer forms affects end joining

**Figure 4.**
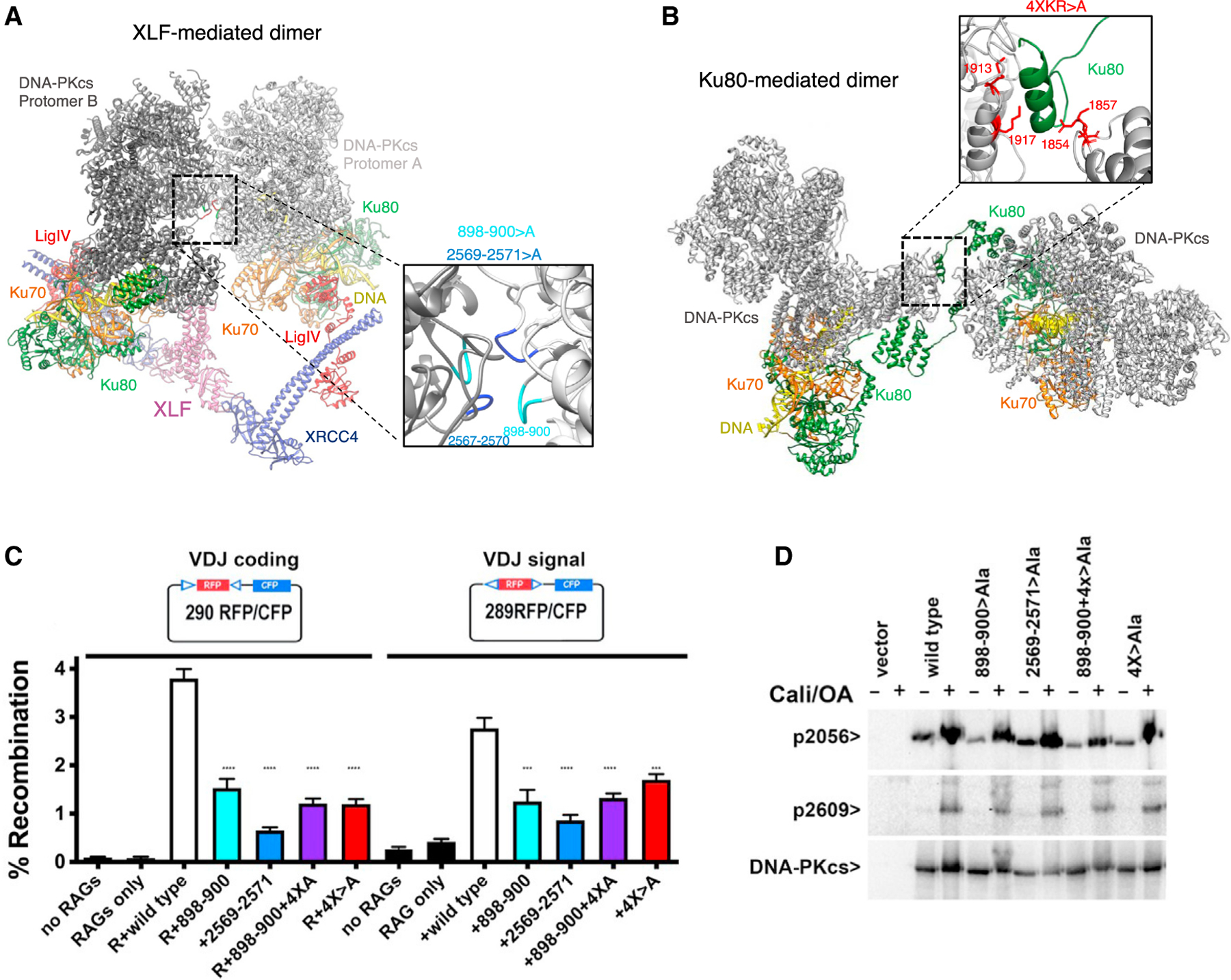
Disruption of DNA-PK dimer interfaces prevents recombination (A) The structure of the NHEJ dimer supercomplex with DNA-PKcs is shown in gray, Ku70 in orange, Ku80 in green, XLF in pink, XRCC4 in blue, DNA in yellow, and LigIV BRCT repeats in red. Inset: enlarged view of the supercomplex dimer interface with L1 (residues 898–900) is shown in cyan and L3 (residues 2567–2570) in blue. (B) The structure of the previous DNA-PK dimer (PDB: 6ZHE) with DNA-PKcs is shown in gray, Ku70 in orange, and Ku80 in green (Chaplin et al., 2021). Inset: enlarged view of the dimer interface, with the four basic residues interacting with the C terminus of Ku80 shown in red. (C) Episomal end-joining assays analyzing the effects of DNA-PKcs dimer interface mutations. DNA-PKcs-deficient V3 cells were co-transfected with the 290-RFP/CFP (red and cyan fluorescent protein) coding joint (left) or 289-RFP/CFP signal joint (right) substrates with no R (RAG1+RAG2), R, or R plus wild-type or mutant DNA-PKcs constructs as indicated. The wild type is shown in white, 898–900 > A (L1) in cyan, 2569–2571 > A (L3) in blue, 898–900 > A and 4XKR > A in purple, and 4XKR > A in red. Cells were analyzed by flow cytometry; the percentage of cells expressing CFP/RFP is indicated as percent recombination. Results were compiled from at least four experiments; ****p < 0.0001, ***p = 0.0002. (D) Immunoblot analyses of whole-cell extracts of DNA-PKcs-deficient 293T cells transiently transfected with no DNA-PKcs, wild-type DNA-PKcs, or mutant DNA-PKcs as indicated. 48 h after transfection, cells were treated with 40 nM calicheamicin and 1 mM okadaic acid for 30 min or left untreated. DNA-PKcs phospho-specific antibodies utilized include anti-phospho-S2056 (working concentration, 1:1000; Abcam 18192) and a rabbit anti-phospho-T2609 reagent, a generous gift from Dale Ramsden (working concentration, 1:500) (Neal et al., 2016).

To address whether dimerization of DNA-PK molecules affects NHEJ, DNA-PKcs expression constructs were designed to introduce alanine mutations that (1) disrupt the XLF-mediated dimeric supercomplex interface presented here (residues 898–900 > A [loop 1] or 2569–2571 > A [loop 3]; Figure 4A); (2) disrupt the interaction between DNA-PKcs and the extreme C terminus of Ku80, part of the domain swap interaction in the DNA-PK dimer reported previously (residues R1854, K1857, K1913, and K1917 mutations 4XKR > A in DNA-PKcs; Figure 4B; Chaplin et al., 2021); or (3) disrupt both dimeric interactions. Unlike other end joining events, VDJ (variability, diversity and joining) recombination is exquisitely dependent on NHEJ. Thus, episomal VDJ recombination assays were performed using the DNA-PKcs-deficient cell strain V3. VDJ coding and signal joining mediated by any of the three mutants is less efficient (by roughly 2-fold) compared with wild-type DNA-PKcs, illustrating the importance of the two types of DNA-PK dimers for efficient NHEJ (Figure 4C). Moreover, combination of the 898–900 > A with the 4XKR > A mutations does not further impair joining (Figure 4C). All four DNA-PKcs mutant constructs were used in transient transfections (in DNA-PKcs-deficient 293T cells); after 48 h, cells were harvested and treated with calicheamicin and okadaic acid. The four DNA-PKcs mutants are expressed similarly, and all undergo calicheamicin-induced autophosphorylation at S2056 and T2609 (Figure 4D), indicating that these mutations do not alter protein expression or phosphorylation levels. This also suggests that phosphorylation of S2056 and T2609 may occur when the dimers are no longer able to form, although, because the dimers are each mediated by several protein/protein interactions, it is possible that the mutants studied here can still form long-range synaptic complexes, albeit inefficiently.

We conclude that formation of DNA-PK dimers facilitates NHEJ. The observation that disruption of both dimer interfaces does not exacerbate end joining deficits suggests that these dimers function in a single NHEJ pathway in which one dimer forms prior to the other dimer or that dimerization facilitates ligation but is not absolutely essential (see the model in Figure 6). It may also be possible that the interplay between the two dimeric forms is more complex and that the importance of one or the other varies under various cellular conditions or in response to specific stimuli.

### Structural rearrangements in the XLF-mediated DNA-PK dimer

**Figure 5.**
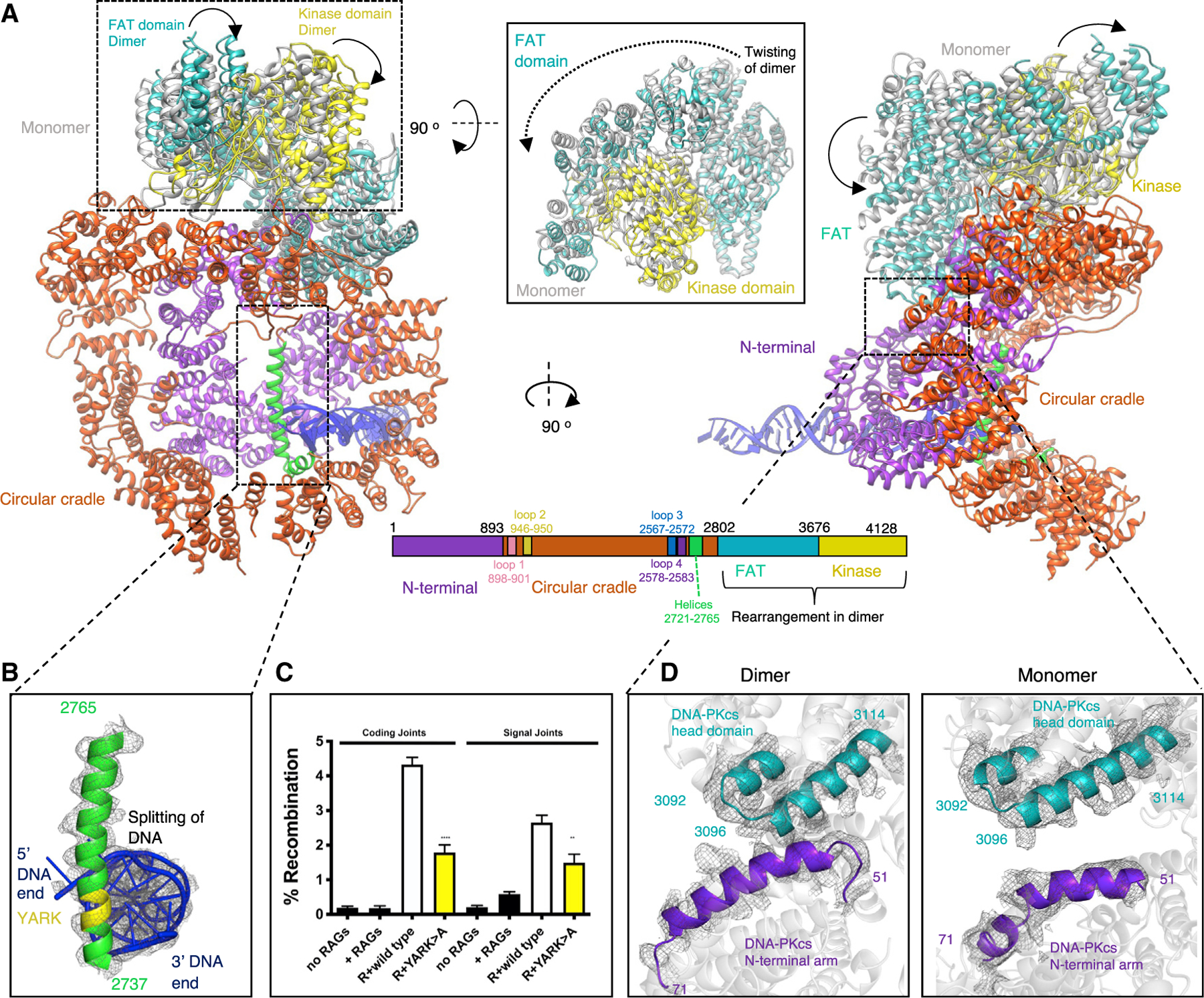
Comparison between DNA-PKcs in the monomeric and dimeric NHEJ supercomplexes (A) Two orientations comparing the structure of DNA-PKcs in the monomer with DNA-PKcs in the dimer. DNA-PKcs in the dimer is colored according to the sequence schematic below the structures, with the N-terminal arm in purple, circular cradle in red-orange, FAT (FRAP [FKBP12-rapamycin-associated protein]) domain in teal, and kinase domain in yellow. DNA-PKcs in the monomer is shown in gray for only the head domain because of the rest of the structure being similar to the dimeric DNA-PKcs structure. The two new helices present in the supercomplex dimeric structure are colored in green and labeled on the sequence schematic. The loops forming the dimer interface in Figure 2 are also labeled and colored on the sequence schematic. Inset: rotation of the head domain by 90° to show the twisting of the head domain in the dimer structure compared with the monomer. (B) A close-up view of the ordered helix (residues 2737–2765) in green and the DNA in blue. (C) Episomal end-joining assays were performed as described in Figure 3. (D) Enlarged view comparing the interaction shown in the dimer (left) between the N-terminal arm shown in purple and the FAT domain in teal with the lack of an interaction shown in the monomer (right). Map density is shown as gray mesh.

**Figure 6.**
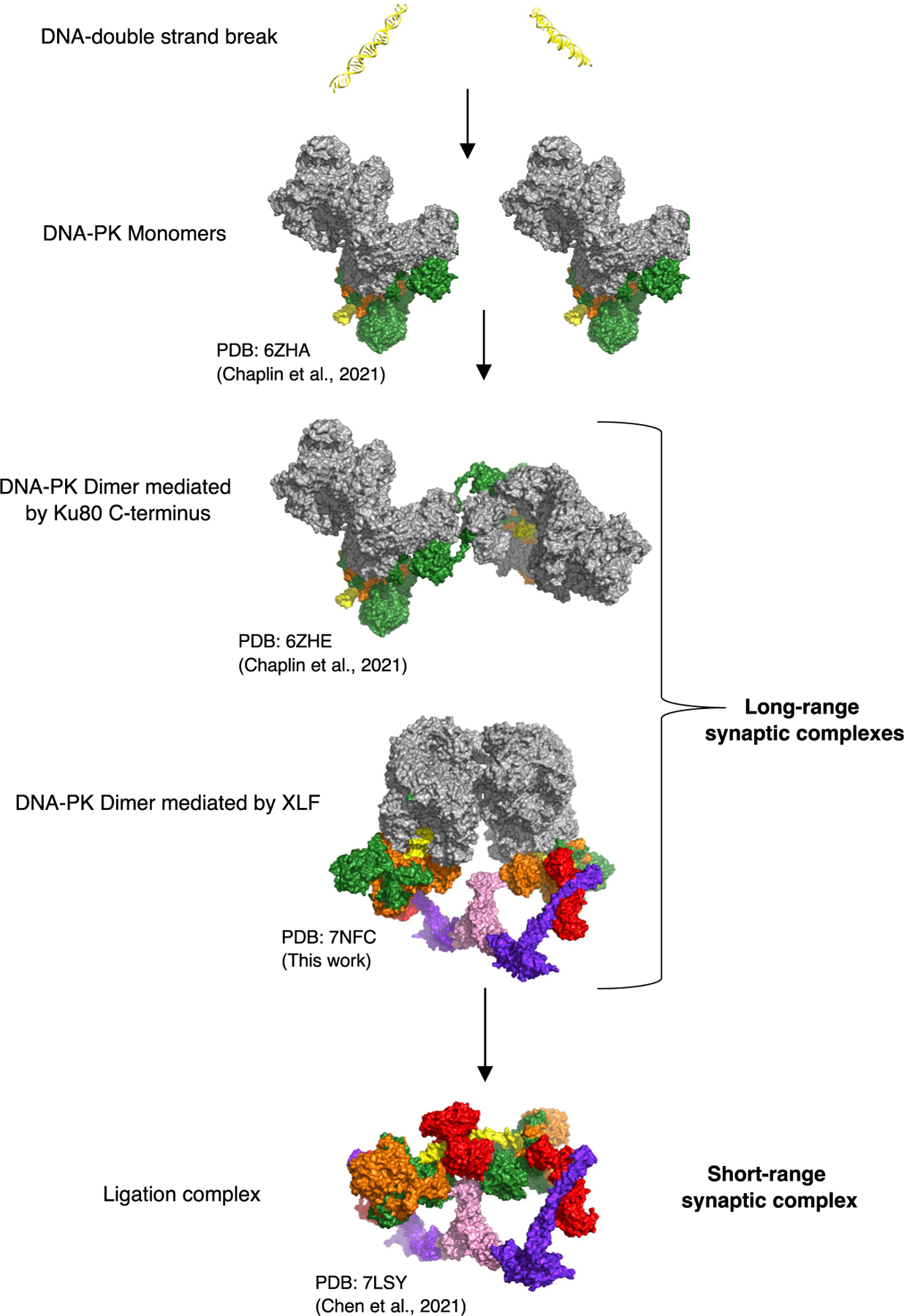
Overall model of NHEJ DNA-PKcs is shown in gray, Ku70 in orange, Ku80 in green, XLF in pink, XRCC4 in blue, DNA in yellow, and LigIV in red. PDB and references are given below the structures.

Within the new dimeric supercomplex form of DNA-PK it is clear that there are major structural rearrangements compared with the monomeric supercomplex structure, with the most notable changes occurring within the head domain of DNA-PKcs. These include a general movement toward the N-terminal arm, resulting in lifting of the FRB (FKBP12-rapamycin-binding) domain toward the dimer interface. In concert with these movements is a twisting of the head domain in relation to the circular cradle (Figure 5A; Videos S1 and S2). Because the movement of the head domain, we now observe a direct interaction between regions encompassing residues 51–71 of the N-terminal arm and 3092–3100 of the head domain (Figure 5C).

In this dimeric supercomplex, we also observe extra density within the circular cradle of DNA-PKcs corresponding to part of the recently postulated plug domain (Hepburn et al., 2021). We have modeled some of this region as residues 2721–2765, which form two new helices. Helix 2738–2765 is positioned down the central cavity of the circular cradle, making a direct interaction with the DNA end, and the second helix, corresponding to residues 2721–2733, packs against helices on the surface of the circular cradle (Figures 5A and 5B). We do not however, observe any density for the remaining ~123 residues (residues 2598–2721) encompassing the disordered region of the ABCDE phosphorylation cluster (residues 2609–2647).

The plug domain has recently been proposed to function as a block on the DNA end. This is in agreement with the positioning of this helix in our structure as it sterically hinders progression of the DNA end within DNA-PKcs (Hepburn et al., 2021). However, in our structure, it appears that the two strands of the DNA are splitting around this helix, with the 5^′^ end of one strand of DNA making interactions with Lys 452 and Tyr 408 and the 3^′^ end of the other strand interacting with Arg 2228 (Figure 5B Video S3). The residues within this helix that directly interact with the DNA ends are residues 2743–2746 (YARK). To address the functional relevance of the DNA end interaction with this helix, a DNA-PKcs expression construct was generated substituting YARK > AAAA. This construct was tested in VDJ episomal end joining assays. Joining is reduced by approximately 2-fold. We conclude that DNA end interaction with this novel helix in the circular cradle facilitates NHEJ. Experiments are underway to assess whether the interaction of the 5^′^ and 3^′^ DNA ends is important for end joining.

## DISCUSSION

In this study, we present monomeric and dimeric NHEJ super-complex cryo-EM structures consisting of DNA-PK, XRCC4, LigIV, and XLF. In these structures, we can see how LigIV, XRCC4, and XLF are able to interact with DNA-PK. An interaction between the BRCT1 domain of LigIV and Ku70/80 has been described previously (Costantini et al., 2007). Our structural data confirm this interaction and allow us to identify specific regions of the BRCT1 domain and the Ku70/80 heterodimer involved in this interaction. Previous reports have inconsistent conclusions regarding whether this interaction is DNA independent (Costantini et al., 2007). Our structure confirms that there are no direct contacts between the BRCT1 domain and DNA; however, it is clear that formation of the Ku70/80 heterodimer is essential to create the BRCT1 domain binding pocket and may enhance stability and, therefore, binding.

The interaction we observe between the stalk of XLF and the vWA domain of Ku70 has not been predicted in earlier work, and this may be due to the interaction being weak or transient in nature. However, the interaction between XLF and the Ku70/80 heterodimer has been localized previously to the vWA domain of Ku80. Interactions between many NHEJ proteins and the Ku70/80 heterodimer have been proposed, predominantly via KBMs, including Werner syndrome protein (WRN), aprataxin- and PNK-like factor (APLF), Cyren (MRI), and PAXX. XLF also contains a KBM at its far C terminus, shown previously to bind to the vWA domain of Ku80, causing an opening in the structure (Nemoz et al., 2018). In our supercomplexes containing XLF, we also observe an opening in the vWA domain of Ku80, which may indicate binding of the C-terminal KBM of XLF (Nemoz et al., 2018).

From our cryo-EM data we were able to reveal a dimeric form of DNA-PK mediated by a central XLF homodimer. Recently, single-molecule fluorescence imaging in *Xenopus laevis* egg extract demonstrates that a single XLF homodimer facilitates assembly of a synaptic complex in NHEJ (Carney et al., 2020; Graham et al., 2018). We observe the central role of XLF in our dimeric form of DNA-PK that would ultimately allow correct positioning of DNA ends prior to ligation. Before our structures presented here and those published very recently (Chen et al., 2021a), such complexes involving NHEJ core proteins have been postulated, but structural data were limited to low-resolution solution scattering and integrative modeling.

Within the dimeric supercomplex structure, there are significant structural rearrangements within the kinase and FAT domain (FRAP [FKBP12-rapamycin–associated protein]) of DNA-PKcs. Recent cryo-EM data have also demonstrated movements within these domains in an active form of DNA-PK (Chen et al., 2021b). The structural rearrangements we observe in our dimeric supercomplex structure are in general agreement with the monomeric DNA-PK complex VI structure from Chen et al. (2021b), in which the kinase domain is reported to be in an active conformation (Figure S6). It should also be noted that, in an additional cryo-EM dataset collected with XLF omitted we also observed movements within the head domain, as seen in the dimeric supercomplex containing XLF. However, in the data with XLF omitted, these structural changes were in conjunction with dimerization of DNA-PK via the C terminus of Ku80 rather than the XLF-mediated dimer form (Figure S7). This suggests that these structural rearrangements are induced by the combination of dimerization and LX4 binding regardless of which dimeric form is assembled. Furthermore, in our XLF-mediated dimeric supercomplex structure, we observe that the PQR and ABCDE phosphorylation clusters appear to be in closer proximity to the kinase domain of the opposite DNA-PK protomer (Figure 2C). However, when this dimer form is mutated, phosphorylation is still detected (Figure 4D). Therefore, although autophosphorylation may occur in *trans* (in the dimeric form), it appears that phosphorylation can also occur in the monomer DNA-PK complex (*cis* conformation). It has been reported previously that *cis* and *trans* phosphorylation can occur (Meek et al., 2007) and that either mechanism may be important for certain steps of the NHEJ pathway (Lu et al., 2008).

One of the most striking features of the new dimeric structure is the ordering of a helix (residues 2737–2765) within the circular cradle of DNA-PKcs. This helix encompasses part of the recently described plug domain (Hepburn et al., 2021). It is clear from our structure that this helix directly contacts the DNA end. It is unclear what the precise role of this helix is, although it appears to act not only as a block for the DNA but also to potentially split the double-stranded DNA, with the two strands separating around the helix and contacting independent sites within DNA-PKcs (Figure 4B). The splitting of the DNA ends around this novel helix suggests a potential mechanism for end processing by DNA-PKcs. It has been shown recently that, although hairpin ends are extremely efficient in promoting DNA-PK’s autophosphorylation of ABCDE sites, hairpin ends do not promote PQR autophosphorylation or phosphorylation of DNA-PK’s many other substrates (Meek, 2020). Several previous studies have suggested that strand separation at the DNA termini enhances DNA-PK’s catalytic activity (Hammarsten et al., 2000; Jovanovic and Dynan, 2006; Pawelczak et al., 2005, 2011; Pawelczak and Turchi, 2008). Moreover, this model would be entirely consistent with work of Graham et al. (2016), who have shown that NHEJ specific end-processing is limited to the short-range complex and that end processing not only requires the presence of core NHEJ factors but also DNA-PK’s catalytic activity. Phosphorylation of the ABCDE sites is the only DNA-PK phosphorylation that has been shown to promote end processing. It should be noted that we used Y-shaped DNA in our cryo-EM samples, and although we believe the Y end is at the end of Ku70/80, it may be possible that the Y end is interacting with the helix and being split either side.

Remarkably, the distance between the DNA ends in the XLF-mediated dimer as presented here and the previously shown Ku80-mediated DNA-PK dimer (Chaplin et al., 2021) are identical at ~115 Å (Figure 3). This distance is in agreement with that proposed from single-molecule FRET experiments to represent a long-range synaptic assembly (Graham et al., 2016). We therefore propose that both of these dimeric forms of DNA-PK represent alternate long-range synaptic complexes. Additionally, our mutational data illustrate the importance of both of these long-range synaptic dimers for efficient end joining. In the XLF-mediated complex, we do not observe density for the catalytic domain of LigIV, potentially because of the flexible linker and inability of the catalytic domain to access the DNA end, precluded by DNA-PKcs. A recent publication has, however, managed to visualize the catalytic domain of LigIV in a low-resolution cryo-EM short-range synaptic complex. In this assembly, where DNA-PKcs was not included, the DNA ends are now able to join together, illustrating the transition of the long-range XLF-mediated dimer to the short-range complex (Chen et al., 2021a). In this short-range synaptic complex, the catalytic domain of LigIV can be visualized, and this may be due to this domain being stabilized by binding of two DNA ends simultaneously (Chen et al., 2021a). This complex also illustrates that the core NHEJ proteins can assemble to form a short-range synaptic complex even in the absence of DNA-PKcs. This may explain why we do not observe an additive effect when both DNA-PK dimers are mutated because synapsis can still occur in the absence of DNA-PKcs (Figure 4). We propose a potential mechanism for NHEJ in which DNA-PK is recruited to the DSB and has the potential to form one of the long-range synaptic dimers (Ku80mediated or XLF mediated) before DNA-PKcs is phosphorylated and removed, allowing the DNA ends to be brought together and the catalytic domain of LigIV to ligate the DNA ends together in the short-range synaptic complex (Chen et al., 2021a).

We demonstrate the fascinating ability of the NHEJ machinery to remodel in response to DSBs. It is striking that, from our structural data, XLF and LX4 make no direct interactions with DNA-PKcs; rather, the interactions with DNA-PK are via the Ku70/80 heterodimer. This allows a scenario where DNA-PKcs could be removed from the assembly to leave a ligation complex composed of only Ku70/80, LX4, and XLF. These structures likely represent only a snapshot of the possible assemblies that could be formed in response to DNA damage. It is well established that multiple additional accessory proteins can be recruited to the NHEJ machinery, and, accordingly, we expect to find different NHEJ supercomplex assemblies with the presence of different accessory factors, processing enzymes, or even DNA-end configurations, highlighting the dynamic character of this pathway.

### Limitations of the study

Although we present monomeric and dimeric NHEJ supercomplex structures and identify new protein-protein interactions, measurement of these interactions using the proteins (such as Ku70 and XLF) in isolation gives undetectable affinities. Therefore, although we show that these interactions form in the context of the supercomplex structure, we cannot show the importance of some of these interactions without the stability of all the proteins and DNA.

## STAR★METHODS

### RESOURCE AVAILABILITY

#### Lead contact

Further information and requests for resources and reagents should be directed to and will be fulfilled by the lead contact, Amanda Chaplin (ac821@cam.ac.uk).

#### Materials availability

Materials supporting the findings of this manuscript are available from the corresponding authors upon reasonable request.

#### Data and code availability

- All data generated or analyzed during this study are included in this published article (and its supplementary information files). Cryo-EM density maps have been deposited in the Electron Microscopy Data Bank and atomic coordinates have been deposited in the RCSB Protein Data Bank. PDB: 7NFE; EMD: 12301 and PDB: 7NFC; EMD: 12299.
- This paper does not report original code.
- Any additional information required to reanalyze the data reported in this work/paper is available from the Lead Contact upon request.

### EXPERIMENTAL MODEL AND SUBJECT DETAILS

DNA-PKcs deficient V3 cells, which is a Chinese hamster ovary cell strain that lacks DNA-PKcs, cultured according to Neal et al. (2016); it was the generous gift of Dr. Martin Gellert, NIH. LX4 was transformed in BL21(DE3) *E. coli* cells and grown in LB media at 37°C, 200 rpm until 0.5 mM IPTG was added and incubated overnight at 16°C, 180 rpm. XLF and Ku70/80 were expressed in SF9 insect cells (Chaplin et al., 2021).

### METHOD DETAILS

#### Purification of DNA-PKcs and Ku70/80

DNA-PKcs and full-length his-tagged Ku70/80 were expressed and purified according to Chaplin et al. (2021).

#### Overexpression and purification of LX4

A vector encoding full-length His-tagged XRCC4-DNA ligase IV (LX4) was transformed into BL21(DE3) *E. coli* cells. Single colonies were picked and grown in LB starter cultures before being transferred into 1 L LB media and grown at 37°C, 200 rpm. Once an OD_600_ of ~0.6 was reached cultures were induced with 0.5 mM IPTG and incubated overnight at 16°C, 180 rpm before harvesting. Cells were harvested by centrifugation (5,020 x g, 20 min, 4°C) and re-suspended in lysis buffer (50 mM Tris, pH 8.0, 5% glycerol (v/v), 150 mM NaCl, 2 mM β-mercaptoethanol, 20 mM imidazole, 10 Protein Inhibitor Cocktail tablets, 20 mg/ml deoxyribonuclease I). LX4 was then purified according to the protocol for Ku70/80 as described according to Chaplin et al. (2021).

#### Expression and purification of full-length XLF

A construct containing full-length 10-His-tagged XLF was expressed in insect cells. Following expression cell pellets were resuspended in lysis buffer (20 mM Tris, pH 8.0, 5%, 50 mM KCl, 50mM NaCl, 5 mM β-mercaptoethanol, 25 mM imidazole, 2 Protein Inhibitor Cocktail tablets per 1 L) and cells sonicated. The resulting lysate was then mixed with 2 µL benzonase (25 kU stock) and MgCl_2_ to a final concentration of 5 mM and left of ice for 20 min. The lysate was then centrifuged (20 min, 30,000 g, 4°C). The supernatant was purified using Ni-NTA resin (QIAGEN) previously equilibrated with lysis buffer and eluted using the lysis buffer containing 300 mM imidazole. Eluted XLF was bound to a Resource Q Sepharose anion exchange column in buffer A (20 mM Tris, pH 8.0, 50 mM KCl, 50 mM NaCl, 5 mM β-mercaptoethanol, 1 mM EDTA) and eluted using a linear gradient of buffer A with 850 mM NaCl. Finally, the protein was dialysed into a final buffer 10 mM Tris, pH 8.0, 150 mM NaCl, 5mM β-mercaptoethanol before being stored at −80°C for further use.

#### DNA annealing

Biotinylated Y-shaped 42–55 bp dsDNA were synthesized and annealed as described previously (Chaplin et al., 2021). DNA sequences used for annealing can be found below.

Y-shaped DNA Forward

Biotin-CGCGCCCAGCTTTCCCAGCTAATAAACTAAAAACTATTATTATGGCCGCACGCGT

Y-shaped DNA Reverse

ACGCGTGCGGCCATAATAATAGTTTTTAGTTTATTGGGCGCG

#### Formation of NHEJ super complexes

Proteins were concentrated using a centricon (Amicon) with a 30 kDa cut-off and buffer exchanged into 20 mM HEPES, pH 7.6, 200 mM NaCl, 0.5 mM EDTA, 2 mM MgCl_2_, 5 mM DTT. Purified Ku70/80 full-length was then mixed with Y-shaped 42–55 bp DNA before being mixed with purified DNA-PKcs, LX4 and XLF in a 2:2:2:2 ratio. The complex was then briefly centrifuged to remove any precipitate/aggregates.

#### Cryo-EM grid preparation

Aliquots of 3 µL of ~2.5 mg/ml of the NHEJ super complex samples were mixed with 8 mM CHAPSO (final concentration, Sigma) before being applied to Holey Carbon grids (Quantifoil Cu R1.2/1.3, 300 mesh), glow discharged for 60 s at current of 25 mA in PELCO Easiglow (Ted Pella, Inc). The grids were then blotted with filter paper once to remove any excess sample, and plunge-frozen in liquid ethane using a FEI Vitrobot Mark IV (Thermo Fisher Scientific Ltd) at 4°C and 95% humidity.

#### Cryo-EM data acquisition

All cryo-EM data presented here were collected on a Titan Krios in the Department of Biochemistry, University of Cambridge and all data collection parameters are given in Table S1.

#### Image processing

13680 movies were collected in accurate hole centering mode using EPU software (Thermo Fisher). CTF correction, motion correction, and particle picking were performed using Warp (Tegunov and Cramer, 2019). 749185 particles picked by boxnet2 masked neural network model in Warp were imported to CryoSPARC (Punjani et al., 2017a, 2017b) for all subsequent processing. These particles were initially subjected to two-dimensional (2D) classification. The initial 2D classes were predominantly of poor visible quality (in part due to the complete random orientation of particles on the grid due to the addition of CHAPSO). However, a small number of classes clearly represented views of DNA-PK, and 25517 particle selected from these classes were used to generate initial *ab initio* 3D volumes representing DNA-PK assemblies. The remaining 723668 particles were also used to generate several *ab initio* 3D volumes to represent particles that do not contain DNA-PK. Particles corresponding to different classes were selected and optimized through multiple iterative rounds of heterogeneous refinement as implemented in CryoSPARC. This process initially used the entire population of picked particles and all initial 3D volumes, and through the iterative process particles not representing DNA-PK assemblies were discarded, while volumes representing DNA-PK were sub-classified to represent various structural states (apo-DNA-PK, super complex monomer, super complex dimer etc.) The process of separating sub-assemblies of DNA-PK was also aided by 3D variability analysis in CryoSPARC (Punjani and Fleet, 2021). The best models were then further refined using homogeneous refinement and finally non-uniform refinement in CryoSPARC. The classification process is summarized schematically in Figure S1. The final reconstructions obtained had overall resolutions (Table S1), which were calculated by Fourier shell correlation at 0.143 cut-off.

#### Structure refinement and model building

The model of the DNA-PK monomer and dimer (PDBs 7NFE and 7NFC) were used as initial templates and rigid-body fitted into the cryo-EM density for the super complexes in UCSF chimera (Pettersen et al., 2004) and manually adjusted and rebuilt in Coot (Emsley et al., 2010). Extra density for LX4 and XLF were docked using PDB 3II6, and PDB 2R9A in UCSF chimera (Pettersen et al., 2004) and again manually adjusted and rebuilt in Coot (Emsley et al., 2010). The new helix in the center of DNA-PKcs and changes in the head domain were remodelled using Coot (Emsley et al., 2010). Namdinator (Kidmose et al., 2019) was used to adjust the final structures and several rounds of real space refinement were then performed in PHENIX (Afonine et al., 2018) before final validation. All structures were refined and validated before being deposited into the PDB with codes shown in Table S1.

#### Episomal end-joining assay

Episomal end-joining assays were performed as described previously (Neal et al., 2016). Briefly, DNA-PKcs deficient V3 cells were co-transfected with either the 290-RFP/CFP coding joint or 289-RFP/CFP signal joint substrates, with either no RAGs, RAGs only, or RAGs plus wild-type or mutant DNA-PKcs constructs. Two mutants were created of the new dimeric supercomplex, loop 1 residues 898–900 to alanine, loop 3 residues 2569–2571 to alanine and one of the previous DNA-PK dimer, where Lys1913, Lys1917, Arg1854 and Arg1857 were all mutated to alanine’s (4XKR > A). We also created a double mutant of 898–900 (loop 1) to ala from the new dimer together with 4XKR > A from the previous dimer. Cells were analyzed by flow cytometry; % of cells expressing CFP/RFP is indicated as % recombination. Results were compiled from at least four experiments; ****p < 0.0001, ***p = 0.0002.

#### Immunoblots

Immunoblot analyses of whole cell extracts of DNA-PKcs deficient 293T cells, transiently transfected with either no DNA-PKcs, wild-type DNA-PKcs, or mutant DNA-PKcs (898–900 > A, 2569–2571 > A, 4XKR > A and 898–900 > A+4XKR > A). 48 hours after transfection, cells were treated or not with 40nM calicheamycin and 1uM okadaic acid for 30 minutes. The DNA-PKcs antibody (working concentration, 1:1000; 42–27) was the generous gift of Tim Carter. DNA-PKcs phospho-specific antibodies utilized include anti-phospho-S2056 (working concentration, 1:1000; Abcam 18192), and a rabbit anti-phospho-T2609 reagent, a generous gift of Dale Ramsden (working concentration, 1:500) (Neal et al., 2016).

### QUANTIFICATION AND STATISTICAL ANALYSIS

Two-tailed unpaired t tests were utilized to compare recombination rates in transfections with wild-type versus mutant DNA-PKcs using Prism9 software, details can be found in Figures 4 and 5 legends.

## Supplementary Material

supplementary data

supplemental video 1

supplemental video 2

supplemental video 3

## Figures and Tables

**Table:** KEY RESOURCES TABLE

REAGENT or RESOURCE	SOURCE	IDENTIFIER
Antibodies		
Anti-DNA PKcs (phospho S2056) antibody	Abcam	ab18192
Bacterial and virus strains		
*E. coli* BL21(DE3) cells	Thermofisher	*EC0114*
*E. coli* Rosetta DE3	Merck	70954
SF9 Insect cells	Oxford Expression Technologies	600100
Chemicals, peptides, and recombinant proteins		
CHAPSO	SIGMA	C3649
Deposited data		
Electron Microscopy Data Bank and the RCSB Protein Data Bank.	This paper	PDB:7NFE; EMD:12301
Electron Microscopy Data Bank and the RCSB Protein Data Bank.	This paper	PDB:7NFC; EMD:12299
Experimental models: Cell lines		
DNA-PKcs deficient V3 cells –The V3 cell line is a Chinese hamster ovary cell strain that lacks DNA-PKcs.	Generous gift of Dr. Martin Gellert	NIH
Oligonucleotides		
Biotin-CGCGCCCAGCTTTCCCAGCTAATAAACTAAAAACTATTATTATGGCCGCACGCGT	Sigma	Yin et al., 2017
ACGCGTGCGGCCATAATAATAGTTTTTAGTTTATTGGGCGCG	Sigma	Yin et al., 2017
Software and algorithms		
CryoSPARC	Punjani et al., 2017a, 2017b	Punjani et al., 2017a, 2017b; https://cryosparc.com
WARP	Tegunov and Cramer, 2019	http://www.warpem.com/warp/#
Coot	Emsley et al., 2010	N/A
UCSF Chimera	Pettersen et al., 2004	N/A
Phenix 1.18	Afonine et al., 2018	N/A

## Highlights

- Cryo-EM of NHEJ supercomplex structures consisting of DNA-PK, XLF, XRCC4, and LigIV
- Description of two dimeric long-range synaptic complexes
- Mutations in these dimeric NHEJ complexes negatively affect DNA repair
- A model of NHEJ consisting of long- and short-range synaptic dimer complexes
