# Supplementary material for: Cryo-EM of NHEJ supercomplexes provides insights into DNA repair
Source: Mol Cell. Author manuscript; Available in PMC 2022 Aug 19. (PMC9006396; doi:10.1016/j.molcel.2021.07.005)
Supplement: supplementary data [file NIHMS1790590-supplement-supplementary_data.pdf]

Molecular Cell, Volume *81*

Supplemental information

Cryo-EM of NHEJ supercomplexes provides insights into DNA repair

Amanda K. Chaplin, Steven W. Hardwick, Antonia Kefala Stavridi, Christopher J. Buehl, Noah J. Goff, Virginie Ropars, Shikang Liang, Taiana Maia De Oliveira, Dimitri Y. Chirgadze, Katheryn Meek, Jean-Baptiste Charbonnier, and Tom L. Blundell

SUPPLEMENTARY:

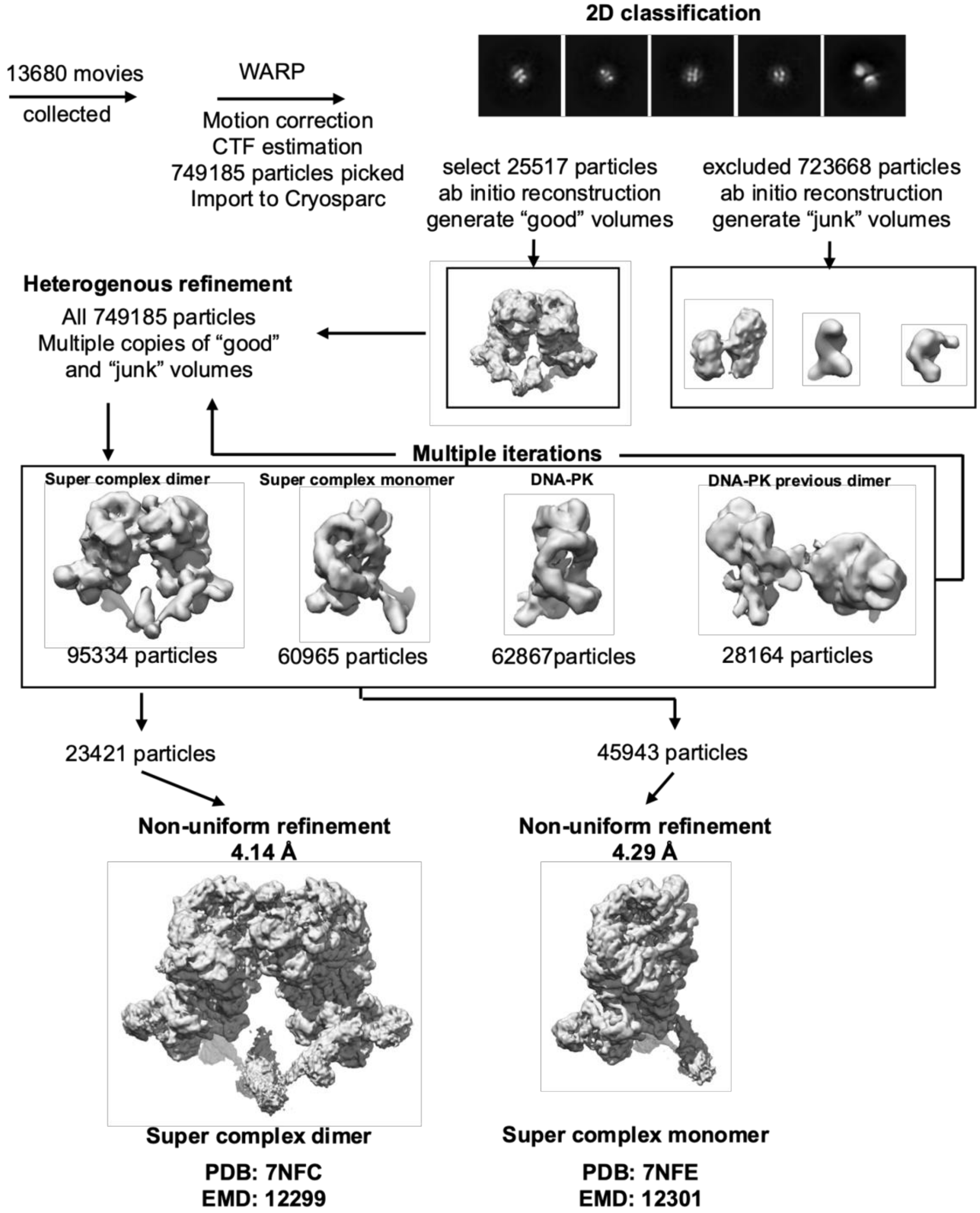

Fig. S1.

Single-particle cryo-EM image processing workflow for the NHEJ super complex structures, related to Figures 1 and 2. Schematic showing particle picking using WARP and processing including 2D classification and ab initio reconstruction using CryoSPARC. The four main classes generated with the corresponding number of particles is shown and the final two super complex maps following non-uniform refinement with resolutions for an FSC of 0.143 are given.

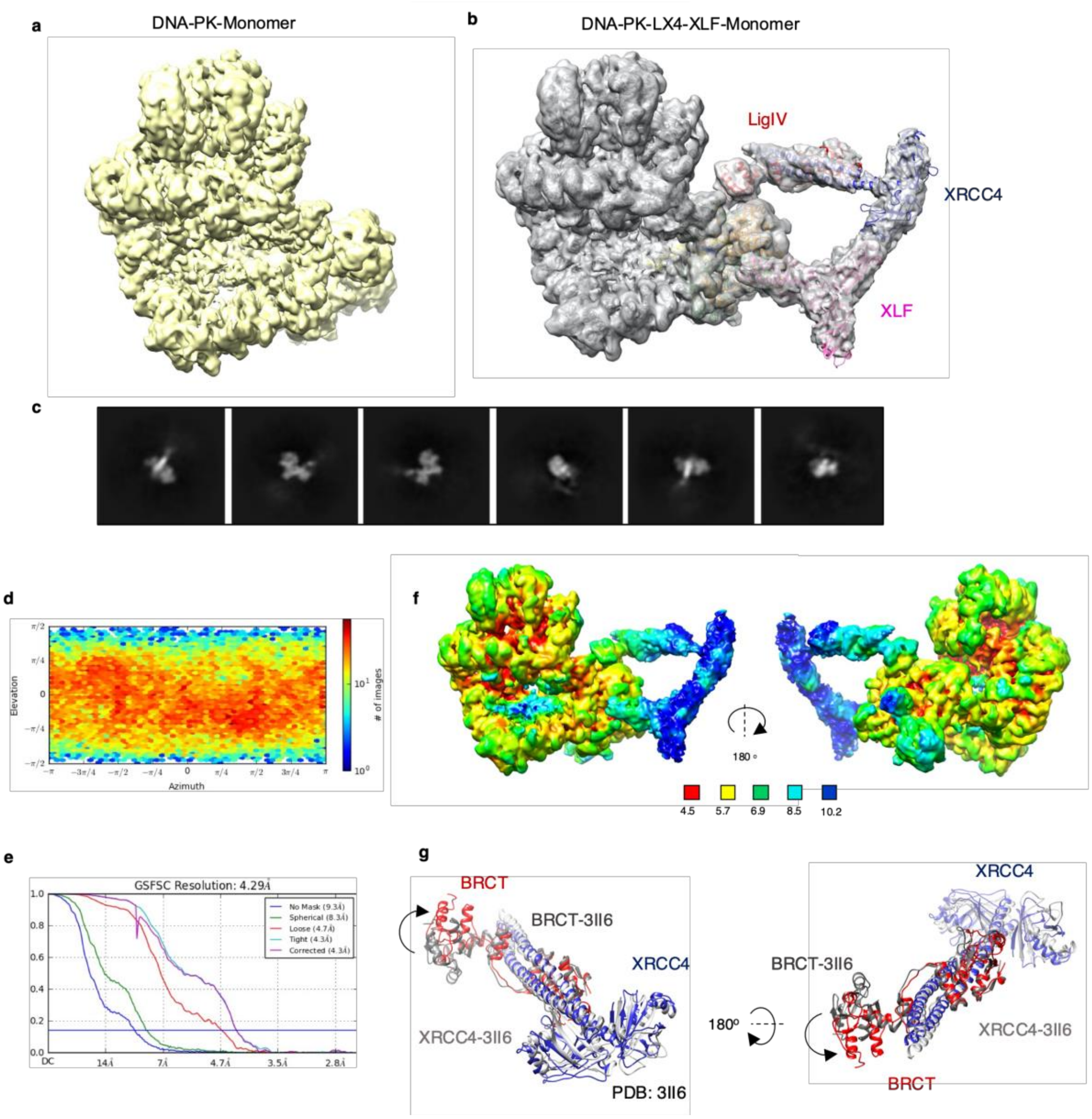

Fig. S2.

Cryo-EM data of the monomeric NHEJ super complex, related to Figure 1. a) Cryo-EM map of the DNA-PK monomer (EMD-11217) **b)** Cryo-EM map of the DNA-PK-LX4-XLF monomeric super complex at 4.3 Å resolution viewed from the same direction as panel a. XLF is shown in pink, XRCC4 in blue, Ku70 in orange and Ku80 in green, DNA-PKcs in grey and the BRCT tandem repeats of LigIV in red with the map shown in transparent. **c)** 2D classes representing the monomeric form of the NHEJ super complex. **d)** Angular distribution calculated in cryoSPARC for particle projections shown as a heat map**. e)** FSC resolution curves and view direction distribution plots of the monomeric form of the NHEJ super complex. **f)** Local resolution map of the DNA-PK-LX4-XLF monomer NHEJ super complex. The map is coloured according to the key showing the colours corresponding to each resolution. **g)** Two orientations comparing XRCC4 (in blue)-LigIV BRCT tandem repeats (in red) from our DNA-PK-LX4-XLF monomer structure with PDB entry 3II6 (grey). Arrows indicate movement between our structure and PDB 3II6.

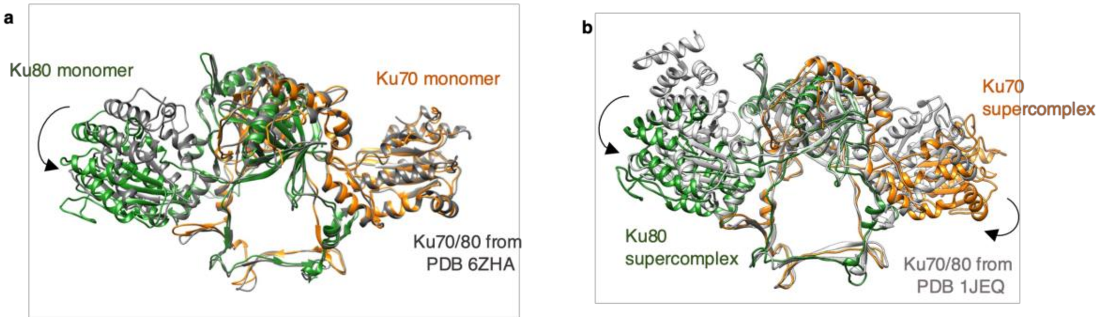

Fig. S3.
Comparison of Ku70/80 structures, related to Figures 1 and 2. a) Ku70/80 (orange and green, respectively) from the monomeric DNA-PK-LX4-XLF NHEJ supercomplex compared with Ku70/80 from the previous DNA-PK monomer (grey, PDB: 6ZHA). An arrow indicates movement in Ku80. **b)** Ku70/80 (orange and green, respectively) from the dimeric DNA-PK-LX4-XLF NHEJ supercomplex compared with Ku70/80 from apo-Ku70/80 (grey, PDB: 1JEQ). An arrow indicates movement in Ku70 and Ku80.

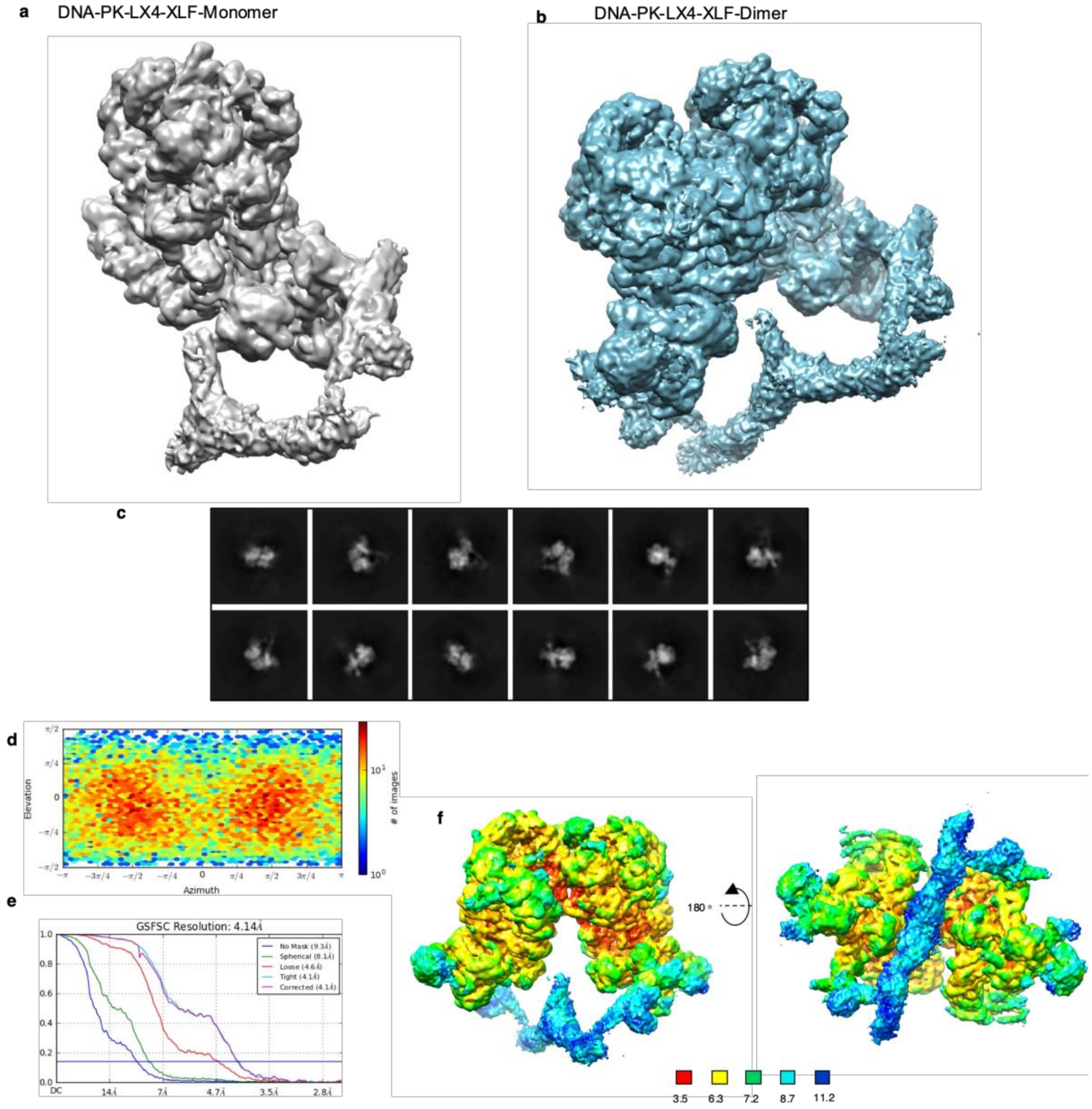

Fig. S4.

Cryo-EM data of the NHEJ super complex dimer, related to Figures 1 and 2. a) Cryo-EM map of the monomeric form of the DNA-PK-LX4-XLF NHEJ super complex at 4.3 Å resolution. **b)** Cryo-EM map of the dimeric form of the DNA-PK-LX4-XLF NHEJ super complex at 4.1 Å resolution. **c)** 2D classes illustrating the dimeric form of the DNA-PK-LX4-XLF NHEJ super complex. **d)** Angular distribution calculated in cryoSPARC for particle projections shown as a heat map. **e)** FSC resolution curves and viewing distribution plot of the dimeric form of the NHEJ super complex.**f)** Local resolution map of the dimeric form of the DNA-PK-LX4-XLF NHEJ super complex. The colours corresponding to each resolution are displayed on the specific key chart below the maps.

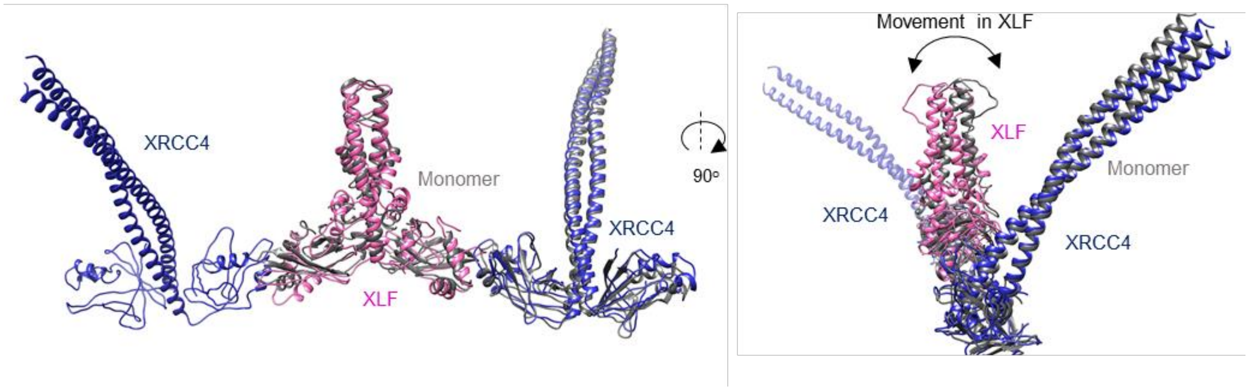

Fig. S5.
Comparison of XLF-XRCC4 between the monomer and dimer DNA-PK-LX4-XLF NHEJ super complex, related to Figures 1 and 2. XRCC4-XLF-XRCC4 filament in the dimer is coloured blue for XRCC4 and pink for XLF and in the monomer XLF-XRCC4 are coloured grey. Movement in the stalk of XLF is shown by arrows.

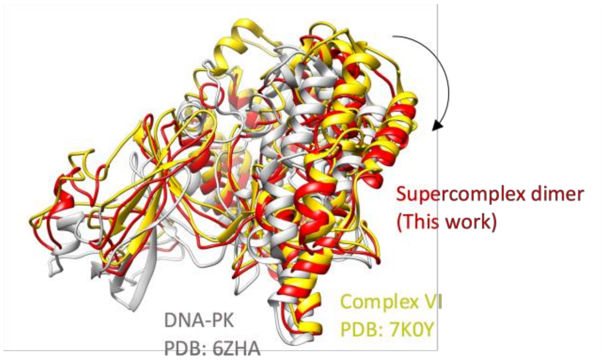

Fig. S6.
Comparison of kinase domains, related to Figure 2. The image displays the kinase domain of DNA-PK (PDB 6ZHA) in grey, the active DNA-PK complex VI (PDB 7K0Y) (Chen el al., 2020), in yellow and the supercomplex dimer from this work in red.

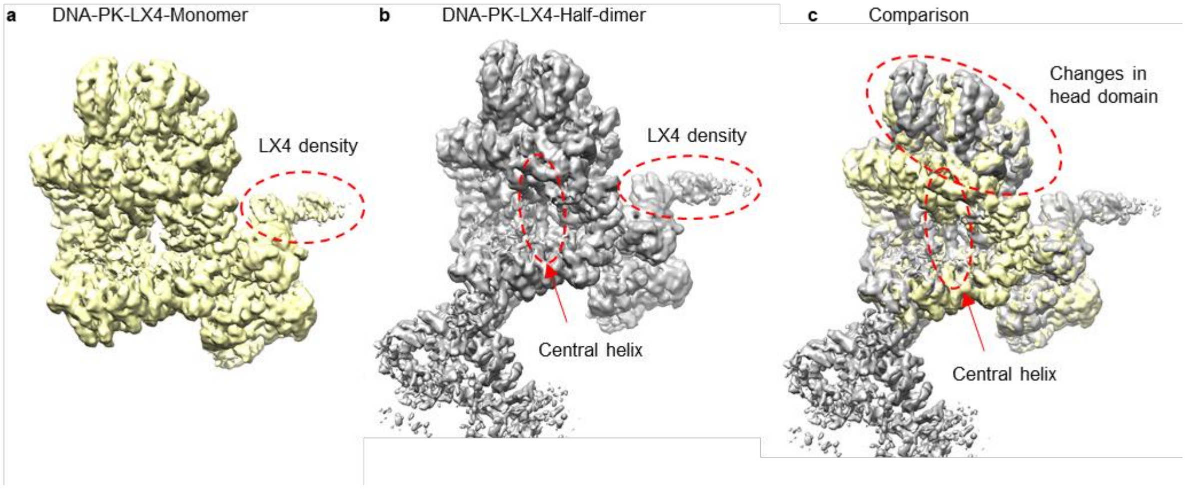

Fig. S7.
Cryo-EM maps of DNA-PK-LX4, related to Figure 2. a) Cryo-EM map of DNA-PK-LX4 monomer to 3.8 Å resolution. Extra density identified for the BRCT tandem repeats of LigIV and the end of the stalk of XRCC4 are circled with a dashed red line. b) Cryo-EM map of DNA-PK-LX4 half dimer to 4.1 Å resolution. Extra density identified for the BRCT tandem repeats of LigIV and the end of the stalk of XRCC4 and the central helix are both circled with a dashed red line. **c)** A comparison between the monomer (yellow) and half dimer (grey) cryo-EM maps of DNA-PK-LX4. Changes in the head domain and appearance of the central helix are circled with a dashed red line.

Table S1: Cryo-EM data collection and refinement statistics, related to Figures 1 and 2.

	DNA-PK Monomer+LX4+XLF PDB:7NFE EMD:12301	DNA-PK Dimer+LX4+XLF PDB:7NFC EMD:12299
**Data collection and processing**		
Detector	Gatan K3	
Magnification	130k	
Energy filter slit width (eV)	20	
Voltage (kV)	300	
Flux on detector (e/pix/sec)	15.186	
Electron exposure on sample (e–/Å^2)	46.8	
Target defocus range (μm)	0.8-2.5	
Calibrated pixel size (Å)	1.304	
Symmetry imposed	C1	

Extraction box size (pixels)	540	
Initial particle images (no.)	749185	
Final particle images (no.)	45943	23421
**Refinement**		
Map resolution at FSC=0.143 (Å)*	4.29	4.14
Model composition		
Non-hydrogen atoms	44758	89160
Protein residues	5623	10927
Nucleotides	48	110
B factor ($Å^2$)		
Protein	361	296.78
DNA	235.6	209.21
R.m.s deviations		
Bond lengths (Å)	0.002	0.003
Bond angles (°)	0.579	0.642
Validation		
Molprobity score	2.02	2.15
Clashscore	14.41	15.89
Poor rotamers (%)	0.11	0.06
Ramachandran plot		
Favored (%)	94.9	93.12
Allowed (%)	5.07	6.81
Disallowed (%)	0.04	0.07
